# ADP secreted by dying melanoma cells mediates chemotaxis and chemokine secretion of macrophages via the purinergic receptor P2Y12

**DOI:** 10.1038/s41419-019-2010-6

**Published:** 2019-10-07

**Authors:** Loreen Kloss, Claudia Dollt, Kai Schledzewski, Andreas Krewer, Susanne Melchers, Calin Manta, Carsten Sticht, Carolina de la Torre, Jochen Utikal, Viktor Umansky, Astrid Schmieder

**Affiliations:** 1 Department of Dermatology, Venereology and Allergology, University Medical Center and Medical Faculty Mannheim, University of Heidelberg, Mannheim, Germany; 2 Center for Medical Research, Medical Faculty Mannheim, University of Heidelberg, Mannheim, Germany; 3 Skin Cancer Unit, German Cancer Research Center (DKFZ), Heidelberg, Germany

**Keywords:** Melanoma, Phagocytes

## Abstract

Melanoma immunotherapy is still not satisfactory due to immunosuppressive cell populations within the tumor stroma. Targeting tumor-associated macrophages (TAM) can help to restore an anti-tumor immunity. Previously, we could show that classical TAM markers expressed in vivo need a 7 day M-CSF/dexamethasone/IL-4 (MDI) stimulation for their induction in peripheral blood monocytes (pBM) in vitro. To identify possible novel therapeutic targets on TAM, gene expression analysis of MDI-treated pBM was performed. This identified up-regulation of the purinergic G-protein coupled receptor P2Y12, the therapeutic target of the clinically approved anti-thrombotic drugs cangrelor, clopidogrel, ticagrelor, and prasugrel. We generated a peptide antibody and validated its specificity using transgenic P2Y12^+^ U937 cells. With the help of this antibody, P2Y12 expression was confirmed on CD68^+^ CD163^+^ TAM of melanoma in situ. Functional analysis revealed that treatment of transgenic P2Y12^+^ U937 cells with the receptor agonist 2-MeSADP induced ERK1/2 and Akt phosphorylation and increased the secretion of the chemokines CXCL2, CXCL7, and CXCL8. These effects could be abolished with the P2Y12 antagonist PSB0739 or with Akt and ERK inhibitors. In addition, P2Y12^+^ macrophages migrated towards the ADP-rich culture medium of puromycin-treated dying B16F1 melanoma cells. Cangrelor treatment blocked migration. Taken together, our results indicate that P2Y12 is an important chemotaxis receptor, which triggers migration of macrophages towards nucleotide-rich, necrotic tumor areas, and modulates the inflammatory environment upon ADP binding.

## Introduction

Immunosuppressive cells such as tumor-associated macrophages (TAM) are prominent components of the tumor microenvironment^1^. A high infiltration of TAM correlates with poor outcome in different tumor entities including melanoma^2^.

In general, macrophages are highly plastic cells that quickly adapt to different environments^3^. Based on in vitro models, macrophages have been classified in pro-inflammatory M1-like and anti-inflammatory M2-like macrophages^4^. While at tumor initiation macrophages predominantly exhibit a M1 phenotype, the tumor microenvironment strongly polarizes macrophages to a M2 phenotype during tumor progression^5^. These TAM promote tumor growth, invasion and angiogenesis by secreting growth factors (e.g. vascular endothelial growth factor) and matrix metalloproteinases. Furthermore, TAM support immune evasion by the expression of various anti-inflammatory mediators such as interleukin 10 (IL-10), transforming growth factor ß (TGF-ß) and arginase-1^6^. By secreting chemokines, they can attract other immunosuppressive cells such as myeloid-derived suppressor cells and regulatory T-cells to the tumor microenvironment^7,8^. Based on the described pro-tumoral and immunosuppressive abilities of M2-like macrophages they classify as promising therapeutic targets especially when combined with immune checkpoint inhibitors^9^. Thus, it is crucial to identify and characterize novel TAM markers that can be targeted therapeutically to improve an anti-melanoma immune response.

P2Y12 is a purinergic, G-protein coupled receptor that is activated by adenosine diphosphate (ADP). Since P2Y12 couples to G_i_ proteins, the Gα subunit inhibits adenylyl cyclase while the Gßγ subunit activates PI3K/Akt signaling^10^. P2Y12 is expressed on platelets, triggering platelet activation and aggregation. Thus, clinically approved P2Y12 antagonists such as clopidogrel, ticagrelor, cangrelor, and prasugrel are widely used to treat thrombotic disorders and are commonly given in combination with aspirin^11^.

Besides platelets, P2Y12 is expressed on microglial cells mediating nucleotide sensing and microglial chemotaxis following nerve injury^12^. Recently, P2Y12 expression was also detected on several other immune cells including dendritic cells, mast cells, and eosinophils^13–15^.

Concerning neoplastic disease, a meta-analysis by the Food and Drug Administration (FDA) in 2014 showed no increased risk in cancer deaths under long-term clopidogrel plus aspirin treatment compared to short-term clopidogrel plus aspirin or aspirin alone^16^. However, some randomized controlled trials reported higher cancer risk especially with ticagrelor and prasugrel^16–18^. Whether these effects derive from the inhibition of platelet aggregation or the blocked binding of ADP to P2Y12 expressed on other hematopoietic cells has not been analyzed yet.

Here we show that P2Y12 is expressed on CD163^+^ TAM of human melanoma in situ. In transgenic P2Y12^+^ U937 cells, ADP induces the secretion of several chemokines among them CXCL2, CXCL7, and CXCL8. Furthermore, we demonstrate that ADP acts as a find-me signal as nucleotides released by dying tumor cells significantly promote the migration of P2Y12^+^ macrophages. Our results indicate that P2Y12^+^ macrophages are attracted to nucleotide-rich tumor environments where they modulate the inflammatory milieu upon ADP binding.

## Results

### Gene expression analysis revealed up-regulation of P2Y12 in pBM_(MDI)_

In the past our group showed that most of the typical human tumor-associated macrophage (TAM) markers such as Stabilin-1, CD163 and Lyve-1 need the combined stimulation with macrophage colony-stimulating factor (M-CSF), dexamethasone and interleukin 4 (IL-4) (MDI) for their induction in human peripheral blood monocytes (pBM) in vitro. To identify possible new therapeutic targets on TAM, we performed gene expression analysis of pBM_(MDI)_ and compared it to M-CSF-stimulated pBM (pBM_(M-CSF)_). One of the highest up-regulated genes in pBM_(MDI)_ was the purinergic receptor P2Y12 (Table 1). Interestingly, P2Y14, which is a member of the P2Y12-like group, was also strongly up-regulated in pBM_(MDI)_ (Table 1). In contrast to P2Y12, this purinergic receptor binds UDP-sugars and plays a role in recruitment of macrophages to the liver^19^. Since P2Y12 is the therapeutic target of already clinically approved anti-thrombotic drugs such as clopidogrel, ticagrelor, cangrelor, and prasugrel and such drugs are not available for P2Y14, we focused on the molecular and functional characterization of P2Y12.

**Table 1:** Gene expression analysis of pBM_(MDI)_ versus pBM_(M-CSF)_

Gene symbol	Gene name	Fold change
P2RY12	Purinergic receptor P2Y, G-protein coupled, 12	44.4
P2RY14	Purinergic receptor P2Y, G-protein coupled, 14	40.8
CCL13	Chemokine (C-C motif) ligand 13	33.9
F13A1	Coagulation factor XIII, A1 polypeptide	31.6
FCER2	Fc fragment of IgE, low affinity II, receptor for (CD23)	30.8
PDK4	Pyruvate dehydrogenase kinase, isozyme 4	28.8
GLDN	Gliomedin	22.9
ADORA3	Adenosine A3 receptor	21.3
CD200R1	CD200 receptor 1	20.1
LYVE1	Lymphatic vessel endothelial hyaluronan receptor 1	15.0
DAAM2	Disheveled associated activator of morphogenesis 2	14.5
EPS8	Epidermal growth factor receptor pathway substrate 8	12.1
SESN1	Sestrin 1	12.0
SEP3	Septin 3	11.7
MAOA	Monoamine oxidase A	11.3
CD209	CD209 molecule	11.2
FGL2	Fibrinogen-like 2	10.2
SHMT1	Serine hydroxymethyltransferase 1 (soluble)	9.5
MTSS1	Metastasis suppressor 1	9.1
SOCS1	Suppressor of cytokine signaling 1	9.1

CD14^+^ pBM were isolated from the blood of three healthy donors and stimulated for 7 days with M-CSF or MDI. Microarray analysis was performed and the gene fold change of MDI versus M-CSF-treated pBM was calculated. Only the 20 highest up-regulated genes are shown

### P2Y12 protein expression can be detected in pBM_(MDI)_

**Fig. 1:**
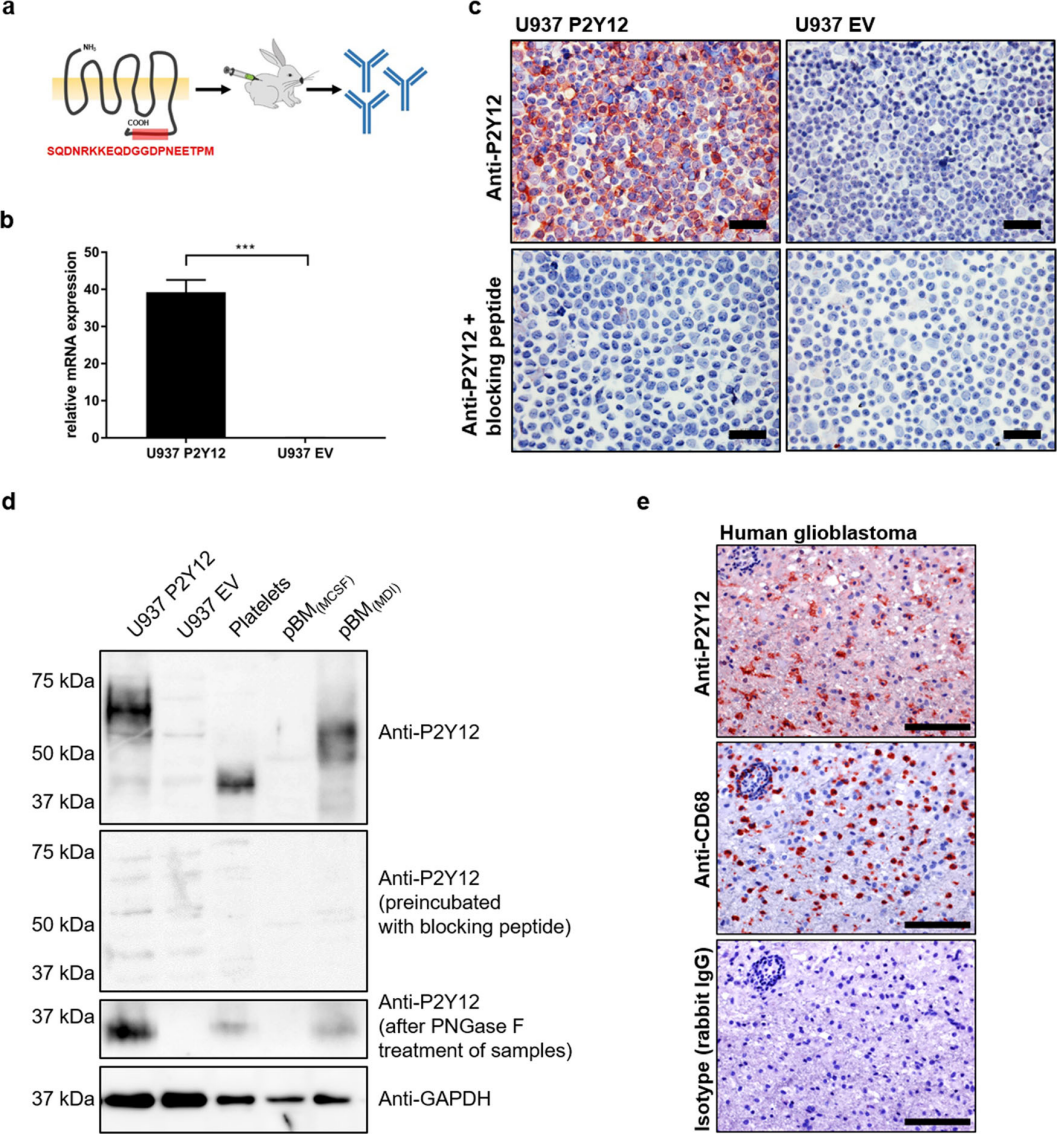
Generation and validation of a specific rabbit anti-hsP2Y12 peptide antibody. **a** A rabbit polyclonal anti-hsP2Y12 antibody targeting the intracellular C-terminal sequence SQDNRKKEQDGGDPNEETPM was generated. **b** P2Y12 mRNA expression in transgenic U937 cells was assessed by qRT-PCR. Gene expression was normalized to ß-actin (*n* = 3). **c** P2Y12 protein expression in transgenic U937 cells was assessed by immunocytochemistry using the self-generated rabbit anti-hsP2Y12 antibody. As a control, the antibody was preincubated with the blocking peptide (SQDNRKKEQDGGDPNEETPM) (*n* = 3, one exemplary experiment is shown). Scale bar = 100 µm. **d** P2Y12 protein expression in transgenic U937 cells, platelets, and pBM was assessed by Western blot analysis using the self-generated rabbit anti-hsP2Y12 antibody. As a control, the antibody was preincubated with the blocking peptide (SQDNRKKEQDGGDPNEETPM). To analyze differences in the N-glycosylation-status of P2Y12 between cells, protein lysates were treated with PNGase F. Anti-GAPDH antibody was used as loading control. **e** Immunohistochemical staining of human glioblastoma sections using anti-hsP2Y12, anti-hsCD68 and isotype control of the anti-hsP2Y12 antibody (rabbit IgG) (*n* = 3). Scale bar = 100 µm. For all stainings one representative picture is shown

To verify protein expression of P2Y12, we generated a rabbit polyclonal anti-hsP2Y12 peptide antibody against the C-terminal amino acid sequence SQDNRKKEQDGGDPNEETPM which is uniquely expressed by P2Y12 and not by other members of the P2Y12-like group (Fig. 1a). Specificity was validated using transgenic P2Y12^+^ U937 cells (Fig. 1b). Immunocytochemical stainings (ICC) and western blot analysis detected P2Y12 exclusively in transgenic P2Y12^+^ U937 cells. Pre-incubation of anti-P2Y12 with the blocking peptide abrogated binding of the antibody to P2Y12 in ICC and western blot analysis (Fig. 1c, d). In platelets our antibody identified a specific protein band at 40 kDa, while in transgenic U937 cells as well as pBM_(MDI)_ the same antibody detected a specific band at ~60 kDa. Both bands have a higher molecular weight than expected for the P2Y12 amino acid sequence (342 amino acids). Since P2Y12 has two described N-linked glycosylation sites (position 6 and 13) we treated the protein lysates with Peptide *N*-Glycosidase F (PNGase F) which led to specific protein bands at ~37 kDa in all three cell types. This corresponds to the actual P2Y12 amino acid length. In addition, immunohistochemical stainings of glioblastoma sections revealed P2Y12 expression in CD68^+^ microglial cells (Fig. 1e) which is in line with already published data by Mildner et al.^20^.

**Fig. 2:**
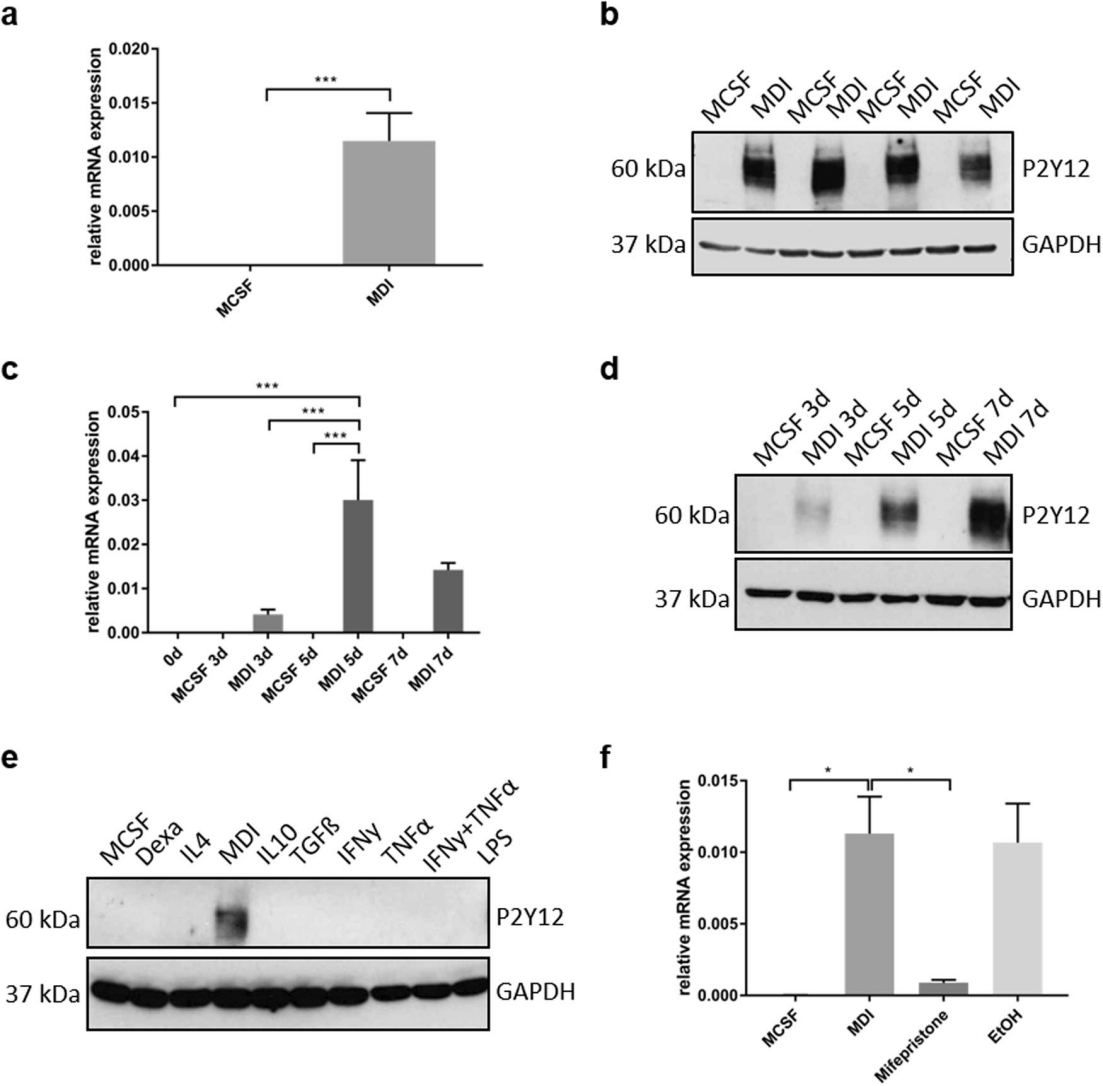
P2Y12 can only be induced by the combined treatment of pBM with MDI. **a**, **b** P2Y12 expression in pBM treated with MDI or M-CSF for 7 days was analyzed by qRT-PCR (**a**) (*n* = 11) and Western blot analysis (**b**) (n = 3). **c**, **d** Time-dependent induction of P2Y12 expression after 3, 5, and 7 days was analyzed by qRT-PCR (**c**) (*n* = 6) and western blot analysis (**d**) (*n* = 3). **e** P2Y12 expression was assessed after treatment of pBM with M-CSF alone or M-CSF plus the indicated pro-and anti-inflammatory cytokines for 7 days by western blot analysis (*n* = 3). **f** P2Y12 induction in pBM treated with M-CSF, MDI, or MDI plus mifepristone for 7 days was assessed by qRT-PCR. Cells treated with equivalent concentration of ethanol (EtOH) were used as control (*n* = 4). For all experiments one representative western blot is depicted. Gene expression was normalized to ß-actin. Data is expressed as mean ± SEM

After the specificity of the antibody was proofed, we used it to correlate protein expression with mRNA levels in pBM. As seen by microarray analysis, MDI strongly induced P2Y12 mRNA which was accompanied by increased protein expression (Fig. 2a, b). P2Y12 induction was time-dependent reaching the highest mRNA levels after five days of stimulation whereas protein expression was even stronger after seven days of MDI treatment (Fig. 2c, d). Stimulations with IL-4 or dexamethasone alone as well as with IL-10, TGF-ß, interferon γ (INF-γ), tumor necrosis factor α (TNF-α), IFN-γ + TNF-α and lipopolysaccharide (LPS) failed to induce P2Y12 expression in pBM (Fig. 2e). Treatment of the cells with the glucocorticoid receptor antagonist mifepristone significantly abrogated the MDI-induced P2Y12 expression (Fig. 2f). Our results indicate that only the combined treatment with MDI induces P2Y12 expression in pBM in vitro. This up-regulation can efficiently be inhibited by the glucocorticoid receptor antagonist mifepristone.

### P2Y12 is expressed on human CD163^+^ TAM of melanoma

**Fig. 3:**
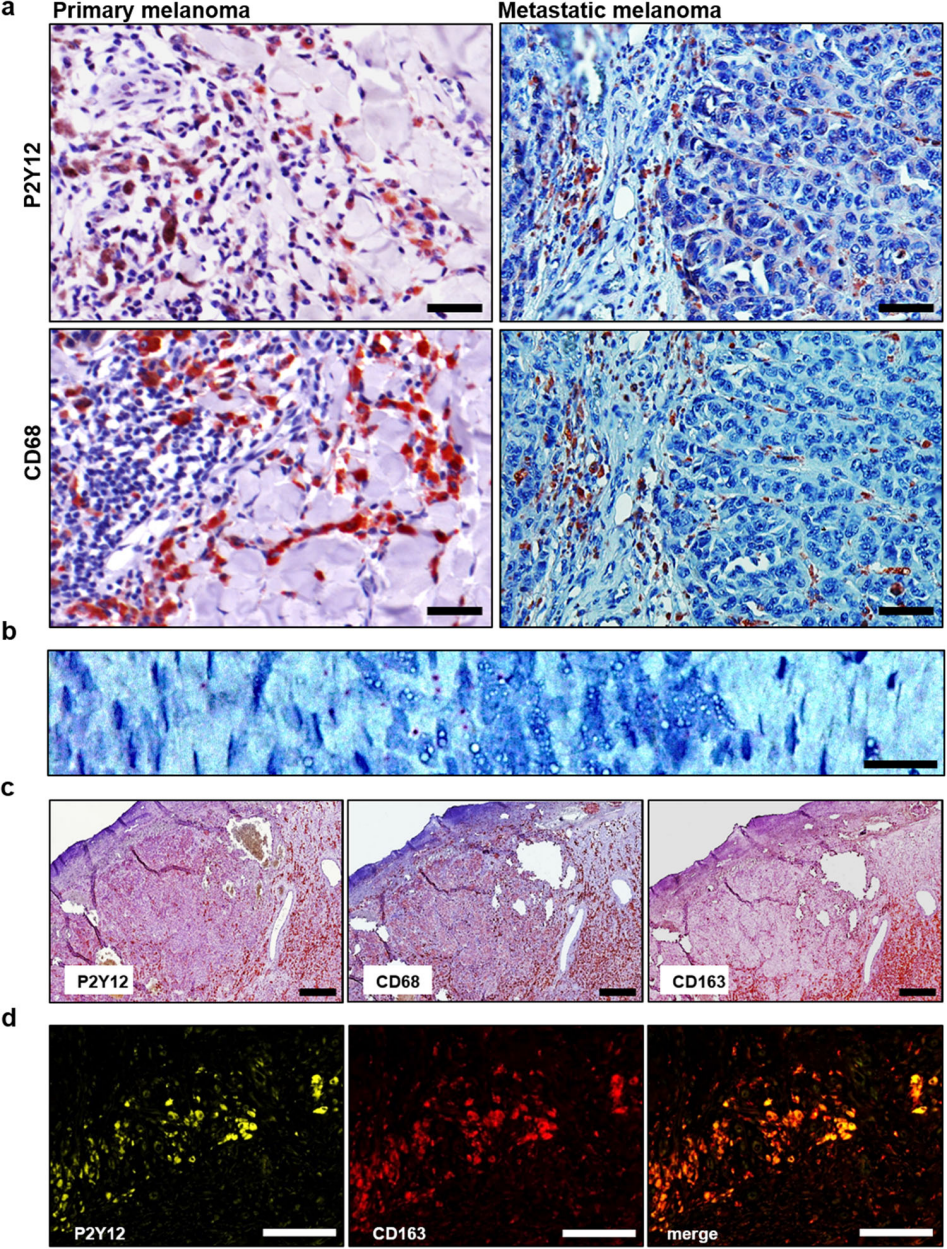
P2Y12 is expressed by human CD163+ TAM of melanoma. **a** Immunohistochemical stainings of human primary as well as metastatic melanoma using anti-hsP2Y12 and anti-hsCD68 antibodies (*n* = 3). **b** In situ hybridization of primary melanoma tissue using a P2Y12-specific probe. Small red dots indicate P2Y12 mRNA (*n* = 3). **c** Sequential staining of one human melanoma section using anti-P2Y12, anti-CD68, and anti-CD163 antibodies (*n* = 2). **d** Immunofluorescent double staining of primary melanoma using anti-CD163 (red)/anti-P2Y12 (green) antibodies (*n* = 3). One representative picture is shown for each staining. Scale bars = 100 µm

Next, we stained human melanoma sections and located P2Y12 on a subset of CD68^+^ macrophages in human primary as well as skin metastatic lesions of melanoma (Fig. 3a). In situ hybridization confirmed this finding on mRNA level (Fig. 3b). Sequential stainings identified P2Y12 on a subpopulation of CD68^+^ TAM which also express CD163 (Fig. 3c). The existence of P2Y12^+^ CD163^+^ TAM was verified by immunofluorescence (Fig. 3d).

### ADP induces the expression and secretion of chemokines in transgenic P2Y12^+^ U937 cells

**Fig. 4:**
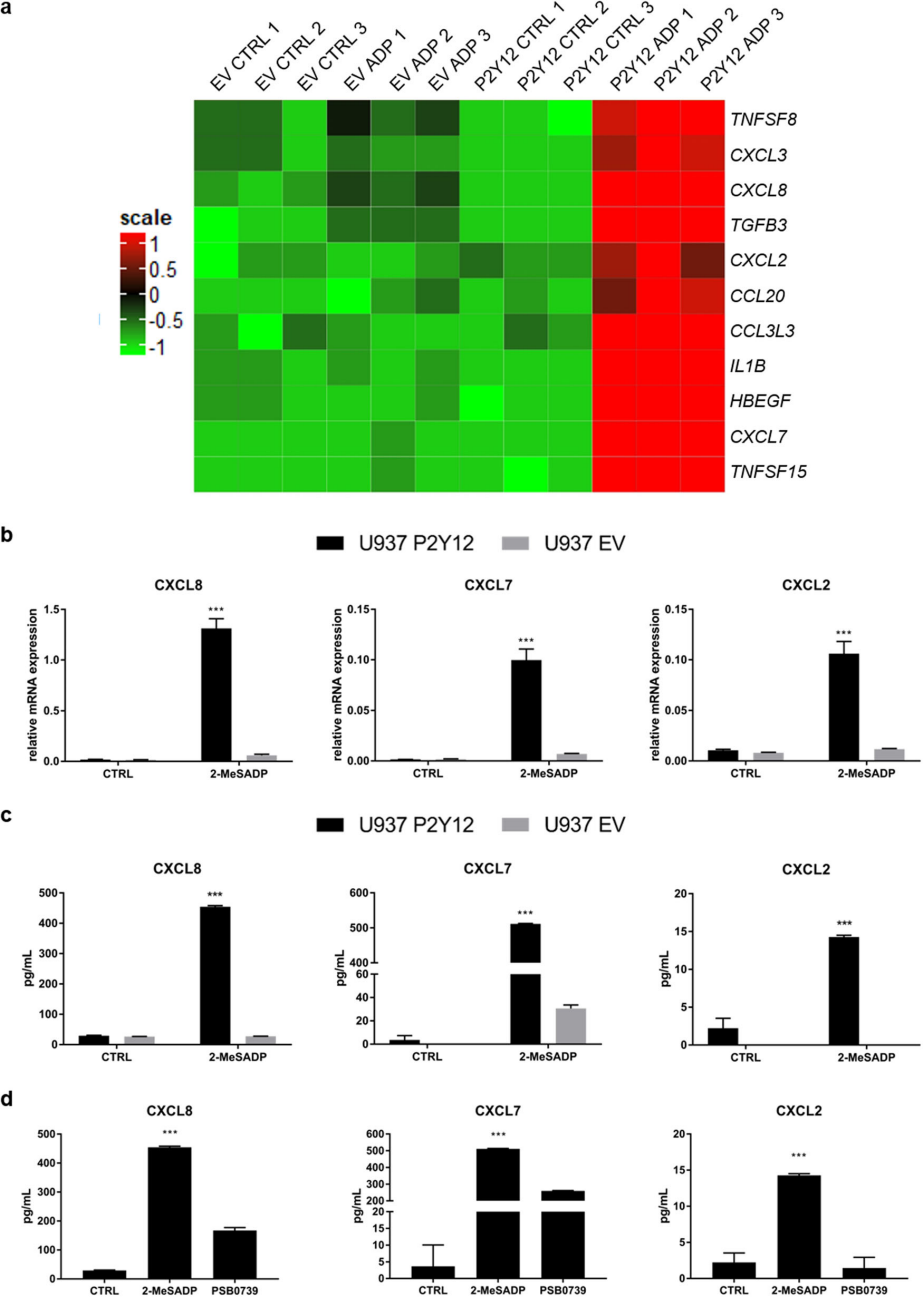
ADP induces chemokine secretion in transgenic P2Y12^+^ U937 cells which can be inhibited with the specific P2Y12 antagonist PSB0739. **a** Gene expression analysis of transgenic U937 cells stimulated with 50 nM 2-MeSADP for 4 h. Heatmap depicts up-regulated cytokines, chemokines, and growth factors in 2-MeSADP-treated P2Y12^+^ U937 cells (P2Y12 ADP) compared to untreated P2Y12^+^ cells (P2Y12 CTRL), 2-MeSADP-treated EV cells (EV ADP), and untreated EV cells (EV CTRL) (*n* = 3). **b** Transgenic U937 cells were treated with 50 nM 2-MeSADP for 4 h and gene expression of the indicated chemokines was assessed by qRT-PCR. Gene expression was normalized to GAPDH (*n* = 4). **c** Chemokine concentrations in the cell supernatants of transgenic U937 cells were assessed by ELISA (*n* = 4). **d** P2Y12^+^ U937 cells were pre-treated with 10 µM PSB0739 prior to stimulation with 50 nM 2-MeSADP for 24 h. Chemokine concentrations in the cell supernatants were determined by ELISA (*n* = 4). Data is presented as mean ± SEM

To identify genes that are up-regulated by ADP in P2Y12^+^ U937 cells, we performed microarray analysis. Instead of ADP we used 2-Methylthioadenosine diphosphate (2-MeSADP) – a stable non-hydrolysable analog of ADP – to avoid cleavage of ADP by ectonucleotidases, which are also known to be expressed by macrophages. Among the highest up-regulated genes in 2-MeSADP-treated P2Y12^+^ U937 cells (P2Y12 ADP) compared to untreated P2Y12^+^ U937 cells (P2Y12 CTRL) were several cytokines [IL-1ß, TGF-ß3, TNF super family member (TNFSF)15, TNFSF8], chemokines (CXCL8, CXCL7, CCL3L3, CXCL3, CCL20, and CXCL2) and growth factors [heparin binding epidermal growth factor (HB-EGF)] (Fig. 4a and Supplementary Table 1). We confirmed up-regulation of the chemokines CXCL8, CXCL7 as well as CXCL2 in P2Y12 ADP by qRT-PCR (Fig. 4b). Elevated concentrations of these chemokines were detected in the supernatant of P2Y12 ADP by enzyme-linked immunosorbent assay (ELISA) (Fig. 4c). Among those chemokines, CXCL7 was the protein with the highest concentration in the supernatant of P2Y12 ADP (511 pg/mL), followed by CXCL8 (454 pg/mL) and CXCL2 (14 pg/mL). When P2Y12^+^ U937 cells were pretreated with the P2Y12-specific antagonist PSB0739, chemokine secretion was significantly reduced (Fig. 4d). We did not find increased chemokine secretion of CXCL2, CXCL8, and CXCL7 in 2-MeSADP-treated pBM_(MDI)_ though, 2-MeSADP up-regulated similar genes in P2Y12^+^ pBM_(MDI)_ as found by microarray analysis in 2-MeSADP-treated P2Y12^+^ U937 cells (data not shown).

### Blockade of ADP-induced ERK and Akt signaling inhibits chemokine secretion in P2Y12^+^ U937 cells

**Fig. 5:**
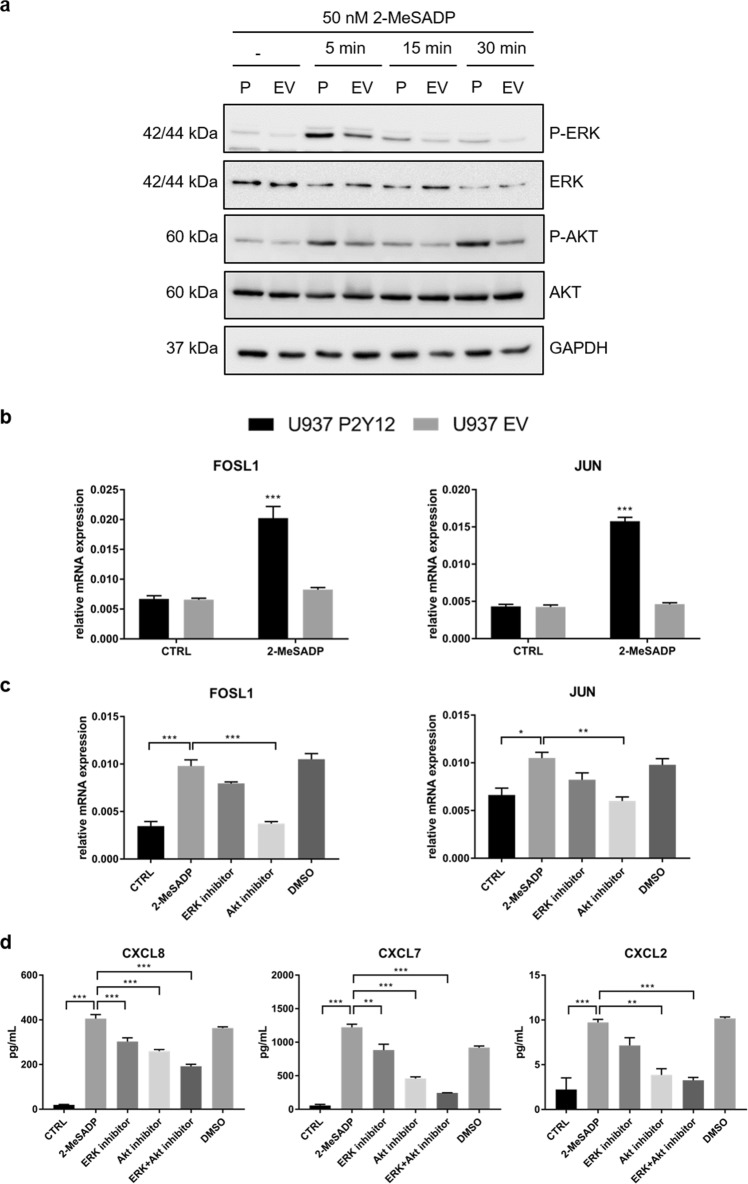
Blockade of ADP-induced ERK and Akt signaling inhibits chemokine secretion in P2Y12^+^ U937 cells. **a** Transgenic U937 cells were treated with 50 nM 2-MeSADP for the indicated time points and western blot analysis was performed using anti-P-ERK1/2, anti-ERK1/2, anti-P-Akt, and anti-Akt antibodies. GAPDH served as loading control. One representative Western blot is shown (*n* = 3). P = P2Y12^+^ U937 cells, EV = empty vector U937 cells. **b** Transgenic U937 cells were treated with 50 nM 2-MeSADP for 4 h and expression of JUN and FOSL1 was assessed by qRT-PCR. Gene expression was normalized to GAPDH (*n* = 3). **c** P2Y12^+^ U937 cells were pretreated with 1 µM ERK inhibitor or 1 µM Akt inhibitor prior to stimulation with 50 nM 2-MeSADP for 4 h. Gene expression of the transcription factors JUN and FOSL1 was assessed by qRT-PCR. Gene expression was normalized to GAPDH (*n* = 3). **d** P2Y12^+^ U937 cells were pretreated with 1 µM ERK inhibitor, 1 µM Akt inhibitor or both prior to stimulation with 50 nM 2-MeSADP for 24 h. Cell supernatants were collected, and chemokine concentrations were determined by ELISA (*n* = 3). Data is presented as mean ± SEM

In order to identify signaling pathways responsible for the ADP-dependent chemokine secretion in P2Y12^+^ U937 cells, we performed western blot analysis. ERK1/2 phosphorylation (after 5 min) as well as AKT phosphorylation (after 5 min and 30 min) was detected after treatment of P2Y12^+^ U937 cells with 2-MeSADP. These signaling pathways were only weakly induced in 2-MeSADP-treated EV cells (Fig. 5a). Furthermore, 2-MeSADP induced the expression of the transcription factor subunits FOSL1 and JUN in P2Y12^+^ U937 cells (Fig. 5b). Pretreatment of these cells with an Akt inhibitor prior to 2-MeSADP stimulation significantly reduced the expression of FOSL1 and JUN while pre-treatment with an ERK inhibitor only slightly decreased the expression of both transcription factor subunits (Fig. 5c). To verify whether 2-MeSADP induces chemokine expression via Akt and/or ERK signaling, we pretreated transgenic P2Y12^+^ U937 cells with Akt and ERK inhibitors and detected reduced secretion of CXCL2, CXCL7 as well as CXCL8 upon 2-MeSADP treatment (Fig. 5d). The strongest inhibition was achieved upon simultaneous blockade of Akt and ERK signaling pathways (Fig. 5d).

### ADP promotes migration of P2Y12^+^ macrophages

**Fig. 6:**
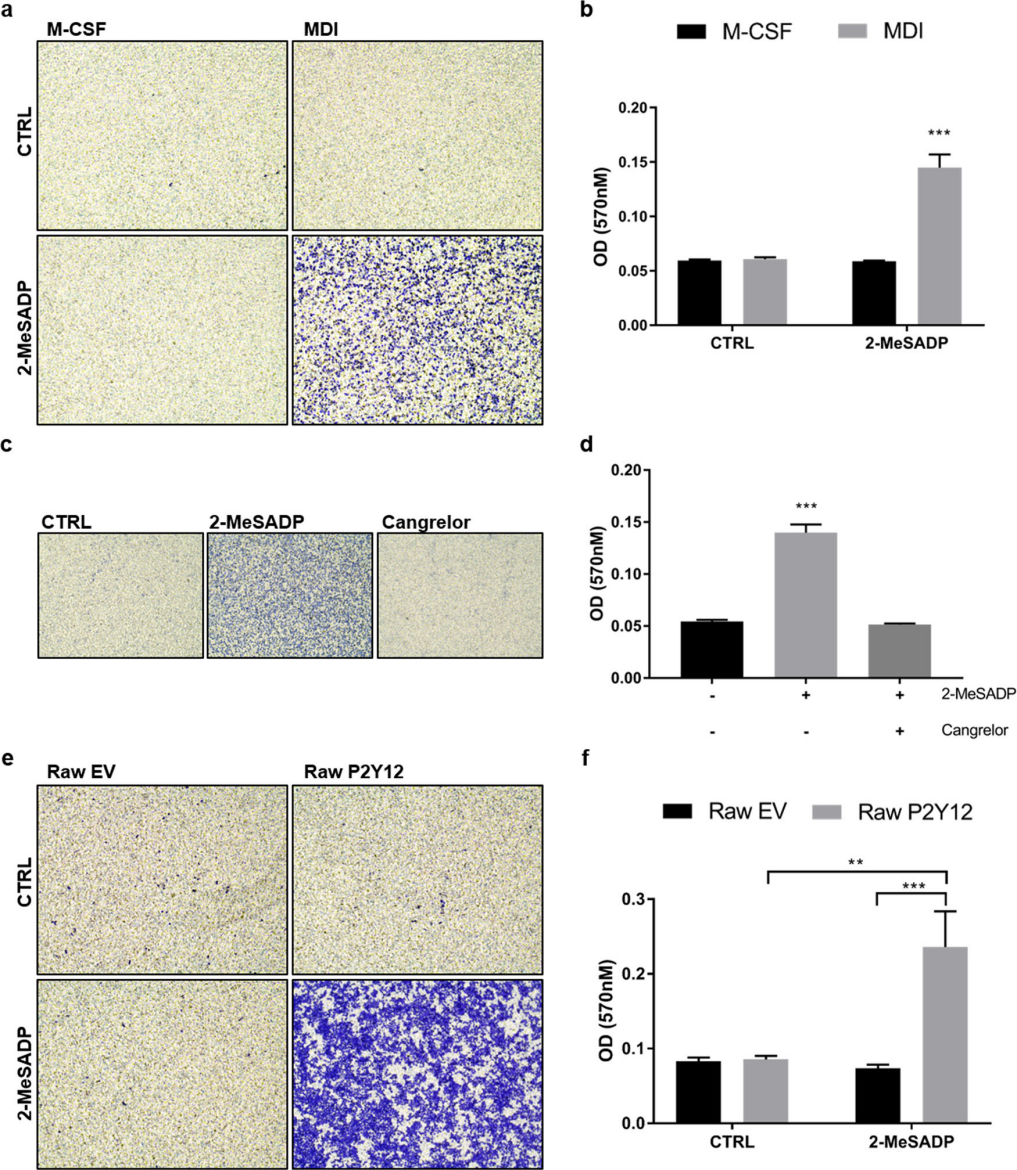
ADP promotes migration of P2Y12^+^ macrophages. **a**, **b** pBM were treated with M-CSF or MDI for 7 days and then seeded in the insert of a transwell chamber. 50 nM 2-MeSADP was added to the medium in the lower chamber. Migration was assessed after 6 h by fixing the cells at the bottom of the transwell membrane with methanol followed by staining with crystal violet. **a** Pictures of the stained migrated cells at the bottom of the transwell membrane were taken using an inverted microscope (*n* = 6). **b** Crystal violet was resolved with methanol and absorbance at 570 nm was measured by microplate reader (*n* = 6). **c**, **d** pBM_(MDI)_ were seeded in the insert of a transwell chamber and 10 µM cangrelor and/or 50 nM 2-MeSADP were added to the medium in the lower chamber. Migration was assessed after 6 h by fixing the cells at the bottom of the transwell membrane with methanol followed by staining with crystal violet. **c** Pictures of the stained migrated cells at the bottom of the transwell membrane were taken using an inverted microscope (*n* = 3). **d** Crystal violet was resolved with methanol and absorbance at 570 nm was measured by microplate reader (*n* = 3). **e**, **f** Transgenic Raw 264.7 cells were seeded in transwell inserts. 50 nM 2-MeSADP was added to the medium of the lower chamber. Migration was assessed after 16 h by fixing the cells at the bottom of the transwell membrane with methanol followed by staining with crystal violet. **e** Pictures of the stained migrated cells at the bottom of the transwell membrane were taken using an inverted microscope (*n* = 6). **f** Crystal violet was then resolved with 100% methanol and absorbance at 570 nm was measured by microplate reader (*n* = 6). Data is presented as mean ± SEM

Since it is known that P2Y12 triggers migration of microglia cells to the site of inflammation, we investigated whether ADP might also act as a chemoattractant for other P2Y12^+^ macrophages. We set up a transwell migration assay with M-CSF and MDI-treated pBM. 2-MeSADP promoted the migration of P2Y12^+^ pBM_(MDI)_ whereas it had no significant effect on the migration of pBM_(M-CSF)_ lacking P2Y12 expression (Fig. 6a, b). The clinically approved P2Y12 antagonist cangrelor inhibited the migration of P2Y12^+^ pBM_(MDI)_ towards 2-MeSADP (Fig. 6c, d). To provide further proof that P2Y12 is responsible for the increased migration of macrophages towards ADP, we used Raw 264.7 cells, an adherent murine macrophage-like cell line, and stably overexpressed P2Y12 in these cells. Like for P2Y12^+^ pBM_(MDI)_, 2-MeSADP also acted as a chemoattractant for P2Y12^+^ Raw 264.7 cells (Fig. 6e, f), while it was no chemoattractant for EV Raw 264.7 cells.

### ADP released by dying tumor cells promotes the migration of P2Y12^+^ Raw 264.7 cells

**Fig. 7:**
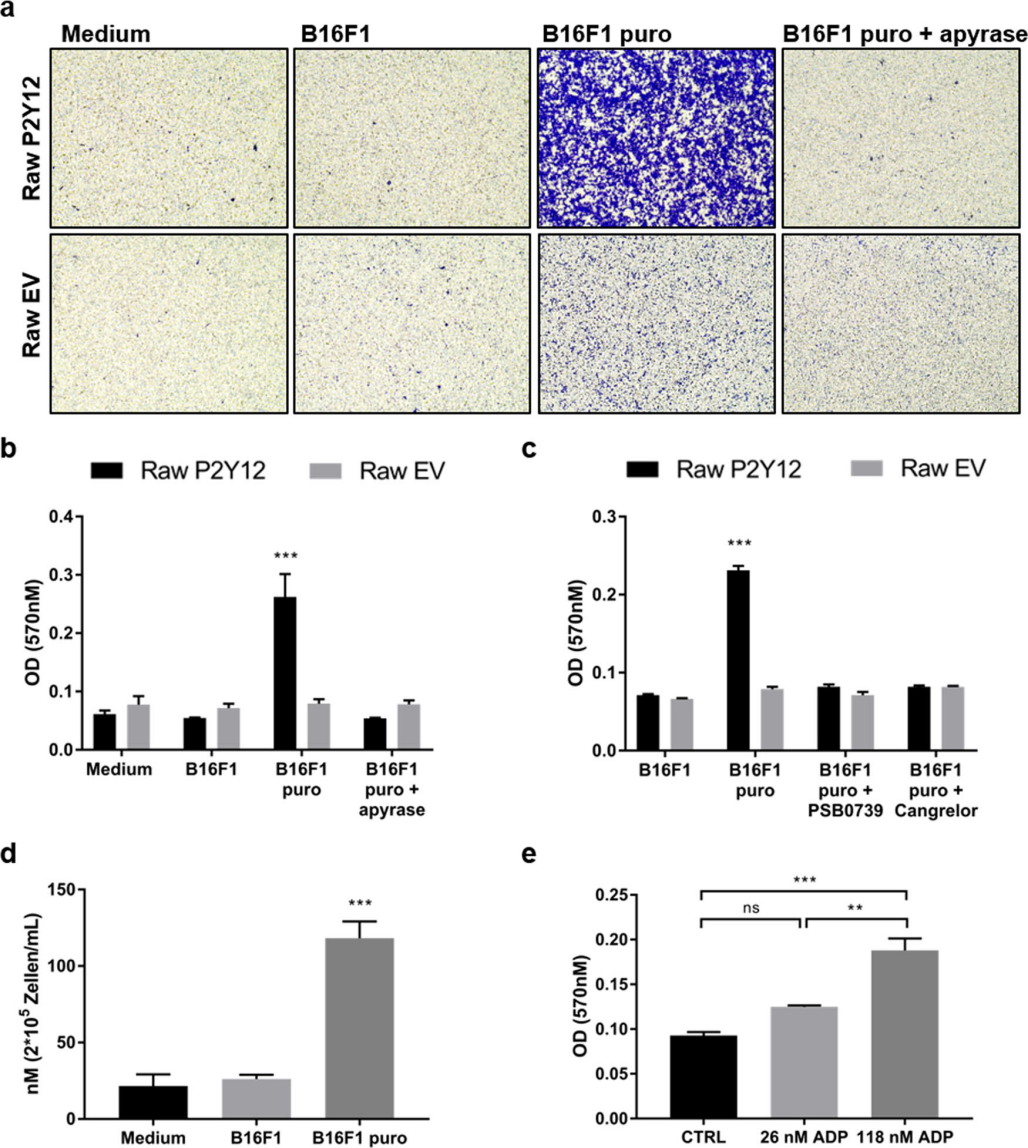
ADP released by dying tumor cells promote the migration of P2Y12^+^ Raw 264.7 cells. **a**–**c** B16F1 were treated with 2 µg/mL puromycin (puro) for 24 h to induce cell death. 1 U/mL apyrase, 10 µM PSB0739, or 10 µM cangrelor were added to the puro-treated B16F1 cells. Untreated B16F1 cells and medium without cells served as controls. Transgenic EV or P2Y12^+^ Raw 264.7 cells were seeded in transwell inserts which were then placed in the 24-well plate containing the B16F1 cells or medium alone. Migration was assessed after 6 h by fixation of the migrated cells with methanol followed by staining with crystal violet. **a** Pictures of the migrated cells at the bottom of the transwell membrane were taken using an inverted microscope (*n* = 6). (**b**–**c**) Crystal violet was then resolved with 100% methanol and absorbance at 570 nm was measured by microplate reader (*n* = 6). **d** Supernatants of untreated and puro-treated B16F1 cells were collected and ADP concentrations were assessed using ADP Assay Kit. Medium was used as a control (*n* = 4). **e** P2Y12^+^ Raw 264.7 cells were seeded in transwell inserts and 26 nM and 118 nM ADP was added to the medium of the lower chamber. Migration was assessed after 6 h by fixing the cells at the bottom of the transwell membrane with methanol followed by staining with crystal violet. Crystal violet was then resolved with methanol and absorbance at 570 nm was measured by microplate reader (*n* = 3). Data is presented as mean ± SEM

To provide evidence that nucleotides released by dying tumor cells act as a find-me signal for P2Y12^+^ macrophages, we set up a transwell co-culture experiment with P2Y12^+^ Raw 264.7 cells and puromycin-pretreated B16F1 melanoma cells. Since the transgenic Raw 264.7 cells contain a puromycin resistance cassette, their viability was not affected by the treatment. Puromycin-pretreated B16F1 cells in the lower transwell chamber significantly promoted the migration of P2Y12^+^ cells, while untreated B16F1 cells as well as medium alone did not (Fig. 7a, b). EV Raw 264.7 cells were only slightly attracted by dying puromycin-pretreated B16F1 cells (Fig. 7a). To test whether the released nucleotides ATP/ADP are responsible for the increased migration of P2Y12^+^ Raw 264.7 cells towards dying tumor cells, we added the ATP/ADP-hydrolyzing enzyme apyrase to the culture medium of the puromycin-pretreated B16F1 cells. Addition of apyrase significantly reduced the migration of P2Y12^+^ Raw 264.7 cells (Fig. 7a, b). Also, treatment of P2Y12^+^ Raw 264.7 cells with the P2Y12 antagonists PSB0739 or cangrelor significantly diminished the migration (Fig. 7c). To determine whether ADP is the cause of increased migration of P2Y12^+^ Raw 264.7 cells towards dying melanoma cells, we measured ADP levels in the cell supernatants of dying and viable B16F1 cells. Increased ADP concentrations were detected in the supernatants of puromycin-treated tumor cells (118 nM ADP) when compared to untreated cells (26 nM ADP) (Fig. 7d). Using these concentrations, we performed transwell migration assays and confirmed increased migration of P2Y12^+^ Raw 264.7 cells towards 118 nM ADP compared to 26 nM ADP (Fig. 7e). These results indicate that dying tumor cells release ADP that attracts macrophages via their P2Y12 receptor.

## Discussion

TAM are prominent components of the tumor microenvironment known to support tumor growth and progression. Therefore, the aim of the current study was to identify novel markers on TAM that can be used as therapeutic targets. Previously, we could show that several M2 macrophage markers expressed by TAM of melanoma in situ can be induced in pBM by MDI in vitro^21^. By gene expression analysis of pBM_(MDI)_, we identified the purinergic receptor P2Y12 as the highest up-regulated gene. On protein level, this ADP receptor has only been verified on platelets and microglia so far but not on other human macrophages including TAM^20,22^. This is probably due to a lack of commercially available specific antibodies targeting human P2Y12. Using our self-generated anti-hsP2Y12 antibody we were the first to identify P2Y12 protein expression in human pBM_(MDI)_ as well as in TAM of melanoma. Various in vitro studies showed that dexamethasone induces an immunosuppressive M2 macrophage phenotype^23^. Although dexamethasone alone was not sufficient to induce P2Y12 expression in pBM, the glucocorticoid receptor inhibitor mifepristone significantly suppressed P2Y12 induction upon MDI treatment indicating that glucocorticoids are indispensable for P2Y12 induction. We detected P2Y12 in CD68^+^ CD163^+^ TAM of primary melanoma as well as melanoma metastases. Not all CD68^+^ macrophages were P2Y12 positive suggesting that this receptor is only expressed on a distinct macrophage subpopulation. Recently, researchers found that glucocorticoid synthesis is also induced in the skin during inflammation or upon exposure to inflammatory stimuli including UV irradiation^24^. Thus, it is possible that local glucocorticoid synthesis in cutaneous melanoma supports macrophage polarization towards an anti-inflammatory M2 phenotype and induces P2Y12. This hypothesis has to be verified using specific knock out mice.

In microglial cells, ADP binding to P2Y12 has been shown to prompt the release of various cytokines and chemokines^25,26^. By gene expression analysis, we also identified up-regulation of several cytokines and chemokines including CXCL2, CXCL7, and CXCL8 in 2-MeSADP-treated P2Y12^+^ U937 cells accompanied by increased protein concentrations in the cell supernatants of these cells. Investigating signaling pathways that lead to the up-regulation of the chemokines in P2Y12^+^ macrophages, we detected both increased ERK1/2 as well as Akt phosphorylation in 2-MeSADP-treated P2Y12^+^ U937 cells as well as an increased expression of the AP-1 transcription factor subunits JUN and FOSL1. In line with our results, P2Y12 agonists induced PI3K/Akt signaling in platelets and microglial cells^27,28^. In addition, Soulet et al.^10^ showed that besides Akt, ERK1/2 is phosphorylated upon treatment of transgenic P2Y12^+^ CHO cells with 2-MeSADP. In our hands, this 2-MeSADP-induced ERK/Akt phosphorylation was responsible for the up-regulation of CXCL2, CXCL7, and CXCL8 as ERK and Akt blockade inhibited the 2-MeSADP-dependent secretion of these cytokines in P2Y12^+^ U937 cells. By releasing these chemokines, P2Y12^+^ macrophages might exert pro-tumoral functions since CXCL2, CXCL7, and CXCL8 are implicated in chemotaxis of neutrophils and in angiogenesis. Since both neutrophil infiltration and angiogenesis support tumor growth and progression, blocking the release of these chemokines with P2Y12 antagonists might attenuate tumor growth. Indeed, treatment of P2Y12^+^ U937 cells with the P2Y12 antagonist PSB0739 abrogated the 2-MeSADP-induced release of CXCL2, CXCL7, and CXCL8. Several clinically approved P2Y12 antagonists including clopidogrel and ticagrelor were reported to have anti-inflammatory properties indicating that P2Y12^+^ cells are important mediators of inflammation. Clopidogrel reduced airway inflammation by decreasing levels of the Th2 cytokines IL-4 and IL-13 as well as IFN-γ and CCL5 in a mouse model of asthma^29^. Ticagrelor attenuated atherogenesis in ApoE-deficient mice. This was accompanied by decreased expression of CCL2 and reduced accumulation of macrophages^30^. In the brain, ticagrelor treatment reduced the number of infiltrating microglia and the expression of IL-1ß, CCL2, and iNOS after cerebral artery occlusion^26^. Together, these results indicate that P2Y12 is involved in the modulation of the immune microenvironment in various pathological inflammatory conditions. In P2Y12^+^ microglia cells ADP does not only induce the secretion of inflammatory mediators but also directly acts as a chemoattractant triggering migration of these cells to the site of nerve injury^12,31,32^. We showed that 2-MeSADP also acts as a chemoattractant for P2Y12^+^ pBM_(MDI)_ as well as P2Y12^+^ Raw 264.7 cells. It is well known that phagocytic cells express purinergic receptors which allow the sensing of nucleotides released by apoptotic and necrotic cells during inflammatory conditions^33^. Zhang et al.^34^ demonstrated that ADP enhanced the recruitment of macrophages and to a lesser extend neutrophils to the site of bacterial infection in vivo suggesting that the ADP receptors P2Y12 and P2Y13 are responsible for this effect. In addition, it was shown that recruited macrophages secrete chemokines such as CXCL2 and CXCL8, which is in line with our findings^35,36^.

Especially in hypoxic areas of rapidly growing tumors such as melanoma the microenvironment is rich in nucleotides released by dying tumor as well as stromal cells^37^. We demonstrated that P2Y12^+^ macrophages migrate towards dying melanoma cells pretreated with puromycin. Since apyrase abrogated this cell death-induced migration and increased ADP levels were detected in the supernatants of dying cells we provided proof that dying tumor cells release ADP that acts as a find-me signal for P2Y12^+^ macrophages. Similar to our findings Elliott et al.^38^ showed that apoptotic cell supernatants induce the migration primarily of macrophages in vivo. Although macrophages of P2Y2^−/−^ mice showed impaired migration towards apoptotic cell supernatants, the migration was not completely abrogated, suggesting the involvement of other nucleotide receptors. Since we showed that P2Y12 is expressed on human TAM and necrotic cell death induces the migration of P2Y12^+^ macrophages, which can be inhibited with the P2Y12 antagonist cangrelor, P2Y12 might also be involved in the sensing of apoptotic cells in the tumor microenvironment. Whether P2Y12 antagonists reduce the migration of P2Y12^+^ macrophages to the site of tumor in vivo and whether P2Y12^+^ macrophages promote tumor growth and progression needs to be determined using appropriate mouse models. Regarding cancer it was shown by others that tumor growth and metastasis are reduced in P2Y12^−/−^ mice^39,40^. In addition, the P2Y12 antagonist ticagrelor reduced metastasis in WT mice^41^. These effects were mainly linked to platelets and not to other P2Y12-expressing hematopoietic cells.

In contrast to these preclinical studies demonstrating an anti-tumoral effect, several clinical trials investigating the effect of dual anti-platelet therapy on cancer incidence concluded that there might be a higher cancer risk with newer anti-platelet drugs such as prasugrel or ticagrelor^16–18^. Whether blockade of specific function of P2Y12^+^ macrophages contribute to this increased cancer risk remains to be elucidated in further studies.

Taken together, our results indicate that P2Y12 is an important immunomodulatory macrophage receptor, which triggers migration of these cells towards ADP-rich tumor areas and is able to modulate the inflammatory environment upon ADP binding.

## Materials and methods

### Cell lines

The human monocytic cell line U937 (CRL-1593.2™, ATCC®, Wesel, Germany) was cultured in RPMI-1640 Medium (Gibco by Thermo Fisher Scientific, Darmstadt, Germany) supplemented with 10% fetal calf serum (FCS, Biochrom, Berlin, Germany), 100 U penicillin, as well as 100 mg/L streptomycin (Pen/Strep, Biochrom, Berlin, Germany). The murine macrophage-like cell line RAW 264.7 (TIB-71™, ATCC®, Wesel, Germany) and the murine melanoma cell line B16F1 (CRL-6323™, ATCC®, Wesel, Germany) were cultivated in DMEM (Gibco by Thermo Fisher Scientific, Darmstadt, Germany) with 10% FCS, 1% Pen/Strep (DMEM complete). All cell lines were cultivated at 37 °C in an atmosphere enriched with 5% CO_2_.

### Isolation of human peripheral blood monocytes

CD14^+^ cells were isolated from buffy coats of healthy donors obtained from Red Cross Blood Service, Baden-Württemberg. Peripheral blood mononuclear cells (PBMC) were separated by density gradient centrifugation and monocytes were isolated by magnetic-activated cell sorting (MACS) using anti-CD14 MicroBeads (Miltenyi, Bergisch-Gladbach, Germany). For differentiation, 1 × 10^6^ cells/mL were seeded in X-VIVO™ 15 medium (Lonza, Cologne, Germany) and stimulated with M-CSF (100 ng/mL, Peprotech, Hamburg, Germany), IL-4 (10 ng/mL, Peprotech, Hamburg, Germany), dexamethasone (1 × 10^−7^ M; 1000 U/mL, Sigma-Aldrich, Munich, Germany), IFN-γ (10 ng/mL, Peprotech, Hamburg, Germany), LPS (1 µg/mL Invitrogen by Thermo Fisher Scientific, Darmstadt, Germany), TNF-α (10 ng/mL, Peprotech, Hamburg, Germany), IL-10 (10 ng/mL, Peprotech, Hamburg, Germany), TGF-ß (100 ng/mL, Peprotech, Hamburg, Germany), and Mifepristone (100 nM, Sigma-Aldrich, Munich, Germany) for 7 days at 37 °C in an atmosphere of 7.5% CO_2_.

### Human samples

The study was performed according to federal laws and regulations as well as institutional policies. We obtained ethical approval from the local ethical committee (reference number: 2010-318N-MA). Written informed consent was obtained from all patients and data was analyzed anonymously.

### Generation of anti-hsP2Y12 antibody

A polyclonal antibody against human P2Y12 was generated in rabbit, targeting the peptide sequence SQDNRKKEQDGGDPNEETPM referring to amino acid 323–342 of the C-terminus (sequence ID NP_073625.1). The synthetized peptide was coupled to KLH carrier protein and used as immunogen in a commercial process (Peptide Specialties Laboratory (PSL), Heidelberg, Germany). The polyclonal anti-hsP2Y12 antibody was purified by affinity chromatography using a peptide specific column (PSL, Heidelberg, Germany).

### Immunocytochemistry

Transgenic U937 cells were centrifuged onto glass slides as cytospins. The slides were washed with PBS, fixed with acetone for 10 min and washed two times with PBS. Subsequently, cytospins were treated with peroxidase blocking solution (Dako by Agilent, Waldbronn, Germany), blocked with 2% BSA, and incubated with anti-hsP2Y12 antibody for 2 h at RT. To test the specificity of the anti-P2Y12 antibody, it was preincubated with the blocking peptide. After incubation with the anti-rabbit HRP-labeled secondary antibody (Dako by Agilent, Waldbronn, Germany) for 1 h at RT, AEC chromogen solution (Dako by Agilent, Waldbronn, Germany) was applied for visualization. 10% Mayer’s Haemalaun (Merck, Darmstadt, Germany) was used for counterstaining. Pictures were taken with a Leica DCRE microscope, Leica DC500 camera, and software system (Leica, Wetzlar, Germany).

### Immunohistochemistry

Formalin-fixed paraffin embedded (FFPE) human tissue samples were dewaxed using a decreasing xylene/alcohol series followed by heat-induced antigen retrieval (pH 6). Subsequently, specimens were treated with peroxidase blocking solution (Dako by Agilent, Waldbronn, Germany), blocked with 2% BSA and incubated with primary antibodies (anti-hsP2Y12 or anti-hsCD68) for 2 h at RT. Rabbit IgG (Dianova, Hamburg, Germany) was used as an isotype control for the anti-hsP2Y12 antibody. After incubation with the appropriate HRP-labeled secondary antibody (for hsP2Y12 anti-rabbit HRP, for hsCD68 anti-mouse HRP, both Dako by Agilent, Waldbronn, Germany) for 1 h at RT, AEC chromogen solution (Dako by Agilent, Waldbronn, Germany) was applied for visualization. 10% Mayer’s Haemalaun (Merck, Darmstadt, Germany) was used for counterstaining. Pictures were taken with a Leica DCRE microscope, Leica DC500 camera, and software system (Leica, Wetzlar, Germany).

### Sequential staining

FFPE melanoma sections were prepared as described before. Samples were blocked with 5% skimmed milk powder in PBS and incubated with the desired primary antibody [self-generated rabbit anti-hsP2Y12, mouse anti-hsCD163 (Leica, Wetzlar, Germany), mouse anti-hsCD68 (Dako by Agilent, Waldbronn, Germany)] diluted in antibody diluent (Dako by Agilent, Waldbronn, Germany) either for 2 h at RT or overnight at 4 °C. The samples were incubated with peroxidase blocking solution (Dako by Agilent, Waldbronn, Germany) followed by incubation with the appropriate HRP-labeled secondary antibody diluted in antibody diluent (Dako by Agilent, Waldbronn, Germany) for 1 h at RT. Samples were incubated with VECTOR NovaRED Peroxidase Solution (Vector laboratories, Peterborough, UK) and counterstained using 10% Mayer’s Haemalaun (Merck, Darmstadt, Germany). After pictures were taken with the Nikon Eclipse NI microscope specimens were destained in stripping buffer [2% SDS, 62.5 mM Tris-HCL (pH 7.5), 0.8% ß-mercaptoethanol] for 1 h at 50 °C and used again to stain another antigen.

### Immunofluorescence

FFPE melanoma sections were prepared as described before. Blocking was performed with 5% donkey serum in PBS. Primary antibodies [self-generated rabbit anti-hsP2Y12 and mouse anti-hsCD163 (Leica, Wetzlar, Germany)] were incubated at 4 °C overnight. Subsequently, the samples were treated with the corresponding fluorochrome-conjugated secondary antibody [donkey anti-rabbit Alexa488 and donkey anti-mouse Cy3 (both Dianova, Hamburg, Germany)]. Images were taken with the Nikon Eclipse NI microscope with the Clara interline CCD camera (Andor, Belfast, UK) and NIS-Elements Advanced software (Nikon, Düsseldorf, Germany).

### In situ hybridization

In situ hybridization was performed with the RNAscope® 2.5 HD Detection Kit (ACDbio, Newark, CA, USA) to visualize P2Y12 mRNA in melanoma specimens. Initially, FFPE tissue samples were deparaffinized with xylene and 100% ethanol and the slides were air-dried. Then, RNAscope® Hydrogen Peroxide (ACDbio, Newark, CA, USA) was applied and the antigen retrieval were performed by boiling in RNAscope® Target Retrieval Reagent (ACDbio, Newark, CA, USA) for 15 min followed by incubation with RNAscope® Protease Plus Reagent (ACDbio, Newark, CA, USA) for 30 min. The P2Y12 probe (Hs-P2Y12 targeting 121–1283, Entrez Gene ID 64805) was hybridized for 2 h at 40 °C and the signal was amplified and detected with RED-A and RED-B solution (ACDbio, Newark, CA, USA) followed by counterstaining with 50% hematoxylin. Pictures were taken with a Leica DCRE microscope, Leica DC500 camera, and software system (Leica, Wetzlar, Germany).

### Western blot analysis

Cells were lysed with RIPA buffer (Sigma-Aldrich, Munich, Germany) containing protease and phosphatase inhibitors (Roche, Mannheim, Germany). For some experiments, denaturated cell lysates were treated with 1 µL PNGase F (Sigma-Aldrich, Munich, Germany) and incubated at 37 °C for 24 h to achieve deglycosylation of the proteins. Proteins were separated by gel electrophoresis using 10% SDS-polyacrylamide gels. After semi-dry blotting (Bio-Rad, Dreieich, Germany) onto PVDF membranes (GE Healthcare, Little Chalfont, UK), the blots were incubated with primary antibodies [self-generated rabbit anti-hsP2Y12, anti-GAPDH, anti-P-ERK1/2, anti-ERK, anti-P-Akt, anti-Akt (Cell Signaling Technology, Danvers, MA, USA)] overnight at 4 °C. To test the specificity of the anti-P2Y12 antibody it was preincubated with the blocking peptide. Subsequently, blots were incubated with the secondary antibodies anti-rabbit HRP (GE Healthcare, Little Chalfont, UK) and for signal detection Luminata Forte Western HRP Substrate (Merck, Darmstadt, Germany) was applied. Signal detection was performed using c600 Western-Blot Imaging System (Azure Biosystems, Dublin, CA, USA).

### Quantitative RT-PCR analysis

RNA was isolated using innuPREP RNA Mini Kit (Jena Analytik, Jena, Germany) according to the manufacturer’s protocol. For reverse transcription 500 ng of RNA per sample was used. The reaction was performed using Maxima Reverse Transcriptase and Oligo(dT)_18_ primer (both Thermo Fisher Scientific, Munich, Germany). Template cDNA was diluted 1:50 in ddH_2_O, mixed with primers (2 μM), as well as SyBRGreen Master Mix (Applied Biosystems by Thermo Fisher Scientific, Munich, Germany) according to the manufacturer’s instructions. The PCR was performed under standard conditions in a MX3000P sequence detection system (Stratagene by Agilent, Waldbronn, Germany). For normalization of the template amount, gene expression was calculated in relation to the housekeeping gene ß-actin or GAPDH. Primers are listed in Supplementary Table 2.

### Generation of transgenic cell lines

Human P2Y12 cDNA (clone IRAUp969F0383D) and murine P2Y12 cDNA (IRAVp968D0977D, both from SourceBioscience, Nottingham, UK) was amplified by PCR and cloned into vector lentivirus ADR3 as described previously^21^. For lentiviral transfection, HEK293/T17 producer cells were transfected with the ADR3 vector carrying the P2Y12 gene and 3rd generation lentiviral plasmids (pMD2.G L1, pRSV rev L2, pMDLg/pRRE L3 and pCDNA3.1/p35 E 71) using X-treme GENE 9 DNA transfection reagent (Roche, Mannheim, Germany). After U937 and RAW 264.7 were infected with the produced lentiviruses positive selection was performed using puromycin (2 µg/mL, Thermo Fisher Scientific, Munich, Germany).

### Enzyme-linked immunosorbent assay

In all, 1 × 10^6^ transgenic U937 cells were seeded in 2 mL RPMI complete and stimulated with 50 nM 2-MeSADP (Bio-Techne, Wiesbaden, Germany) for 24 h. Cell supernatants were collected and centrifuged at 1000 × *g* for 10 min to obtain cell free supernatants. All ELISAs were performed according to the manufacturer’s instructions. (human CXCL8/CXCL2/CXCL7 DuoSet ELISA, R&D Systems, Wiesbaden, Germany).

### Microarray analysis

Transgenic U937 cells were seeded at a concentration of 1 × 10^6^ cells/mL and stimulated with 50 nM 2-MeSADP (Bio-Techne, Wiesbaden, Germany) for 4 h. pBM were seeded at a concentration of 1 × 10^6^ cells/mL and stimulated with M-CSF and MDI for 7 days as described before. Gene expression profiling was performed using arrays of human HuGene-2_0-st-type (Thermo Fisher Scientific, Waltham, MA, USA). Biotinylated antisense cDNA was then prepared according to the standard labeling protocol with the GeneChip® WT Plus Reagent Kit and the GeneChip® Hybridization, Wash and Stain Kit (both from Thermo Fisher Scientific, Waltham, MA, USA). Afterwards, the hybridization on the chip was performed on a GeneChip Hybridization oven 640, then dyed in the GeneChip Fluidics Station 450 and thereafter scanned with a GeneChip Scanner 3000. All of the equipment used was from the Affymetrix-Company (Affymetrix, High Wycombe, UK). A Custom CDF Version 20 with ENTREZ based gene definitions was used to annotate the arrays^42^. The raw fluorescence intensity values were normalized applying quantile normalization and RMA background correction. OneWay-ANOVA was performed to identify differential expressed genes using a commercial software package SAS JMP10 Genomics, version 6, from SAS (SAS Institute, Cary, NC, USA). A false positive rate of *a* = 0.05 with FDR correction was taken as the level of significance. Gene Set Enrichment Analysis (GSEA) was used to determine whether defined lists (or sets) of genes exhibit a statistically significant bias in their distribution within a ranked gene list using the software GSEA^43^.

### Transwell migration assay with pBM

CD14^+^ cells were isolated and differentiated as described before. After seven days of stimulation, MDI- and M-CSF-treated pBM were harvested and 2 × 10^5^ cells were seeded in 6.5 mm transwell inserts with a 5-µm pore size (Corning, Wiesbaden, Germany). X-VIVO medium supplemented with 50 nM 2-MeSADP (Bio-Techne, Wiesbaden, Germany) was used as a chemoattractant in the lower chamber of the transwell. For some experiments pBM_(MDI)_ were pretreated with 10 µM cangrelor (Bio-Techne, Wiesbaden, Germany). Migration was assessed after 6 h by fixing the cells with 100% methanol, followed by staining with 5% crystal violet. Pictures of migrated cells were taken using an inverted microscope (Zeiss Axiovert). Crystal violet was then dissolved in methanol and quantified measuring the absorbance at 570 nm by TECAN microplate reader.

### Transwell migration assay with transgenic Raw 264.7 cells

In all, 5 × 10^5^ transgenic Raw 264.7 cells were seeded in DMEM w/o FCS in the upper chamber of a 6.5-mm transwell insert with a 5-µm pore size (Corning, Wiesbaden, Germany). DMEM complete supplemented with 50 nM 2-MeSADP (Bio-Techne, Wiesbaden, Germany) was used as a chemoattractant in the lower chamber of the transwell. Migration was assessed after 16 h as described before. For migration experiments with dying tumor cells 2 × 10^4^ B16F1 melanoma cells were seeded in 24-well plates and cell death was induced with 2 µg/mL puromycin for 24 h. Transwell inserts loaded with 5 × 10^5^ transgenic Raw 264.7 cells in 100 µL DMEM were added to the 24-well plate containing the puromycin-treated B16F1 cells. Untreated B16F1 cells and medium only served as controls. Either 1 U/mL apyrase was added to the puromycin-treated B16F1 cells or transgenic Raw 264.7 cells were pre-treated with 10 µM of the P2Y12 antagonists PSB0739 and cangrelor (both from Bio-Techne, Wiesbaden, Germany). For distinct experiments, ADP (Sigma-Aldrich, Munich, Germany) instead of 2-MeSADP was added to the lower chamber of the transwell. Migration was assessed after 6 h as described before.

### ADP assay

In all, 2 × 10^4^ B16F1 melanoma cells were seeded in 24-well plates and cell death was induced with 2 µg/mL puromycin for 24 h. Cell supernatants were harvested and ADP concentration was determined using ADP Assay Kit (Sigma-Aldrich, Munich, Germany).

### Statistics

Statistical analyses of all data were calculated by using GraphPad Prism 6.0 (GraphPad Software, USA). Statistical significance was assessed by using Student’s *t*-test or by one-way ANOVA and Bonferroni as a post-test. The level of significance is indicated by asterisks (*** ≤ 0.001; ** ≤ 0.01 and * ≤ 0.05). Error bars depict standard error of mean (SEM) of each experiment. All experiments were performed at least in triplicates.

## Supplementary information

Supplemental Data

## References

[1] Gajewski TF, Schreiber H, Fu YX (2013). Innate and adaptive immune cells in the tumor microenvironment. Nat. Immunol..

[2] Falleni M (2017). M1 and M2 macrophages’ clinicopathological significance in cutaneous melanoma. Melanoma Res..

[3] Biswas SK, Chittezhath M, Shalova IN, Lim JY (2012). Macrophage polarization and plasticity in health and disease. Immunol. Res..

[4] Mills CD, Kincaid K, Alt JM, Heilman MJ, Hill AM (2000). M-1/M-2 macrophages and the Th1/Th2 paradigm. J. Immunol..

[5] Schmieder A, Michel J, Schönhaar K, Goerdt S, Schledzewski K (2012). Differentiation and gene expression profile of tumor-associated macrophages. Semin. Cancer Biol..

[6] Biswas SK, Allavena P, Mantovani A (2013). Tumor-associated macrophages: functional diversity, clinical significance, and open questions. Semin. Immunopathol..

[7] Gabrilovich DI, Ostrand-Rosenberg S, Bronte V (2012). Coordinated regulation of myeloid cells by tumours. Nat. Rev. Immunol..

[8] Ostrand-Rosenberg S, Sinha P, Beury DW, Clements VK (2012). Cross-talk between myeloid-derived suppressor cells (MDSC), macrophages, and dendritic cells enhances tumor-induced immune suppression. Semin. Cancer Biol..

[9] Cassetta Luca, Pollard Jeffrey W. (2018). Targeting macrophages: therapeutic approaches in cancer. Nature Reviews Drug Discovery.

[10] Soulet C (2004). Gi-dependent and -independent mechanisms downstream of the P2Y12 ADP-receptor. J. Thrombosis Haemost..

[11] Cattaneo M (2015). P2Y12 receptors: structure and function. J. Thromb. Haemost..

[12] Haynes SE (2006). The P2Y12 receptor regulates microglial activation by extracellular nucleotides. Nat. Neurosci..

[13] Ben Addi A, Cammarata D, Conley PB, Boeynaems JM, Robaye B (2010). Role of the P2Y12 receptor in the modulation of murine dendritic cell function by ADP. J. Immunol..

[14] Muniz VS (2015). Purinergic P2Y12 receptor activation in eosinophils and the schistosomal host response. PLoS ONE.

[15] Paruchuri S (2009). Leukotriene E4-induced pulmonary inflammation is mediated by the P2Y12 receptor. J. Exp. Med..

[16] Kaufmann CC, Lyon AR, Wojta J, Huber K (2019). Is P2Y12 inhibitor therapy associated with an increased risk of cancer?. Eur. Heart J. Cardiovasc. Pharmacother..

[17] Kotronias RA (2017). Cancer event rate and mortality with thienopyridines: a systematic review and meta-analysis. Drug Saf..

[18] Leader A (2017). The effect of combined aspirin and clopidogrel treatment on cancer incidence. Am. J. Med..

[19] Xu J (2012). GPR105 ablation prevents inflammation and improves insulin sensitivity in mice with diet-induced obesity. J. Immunol..

[20] Mildner A, Huang H, Radke J, Stenzel W, Priller J (2017). P2Y12 receptor is expressed on human microglia under physiological conditions throughout development and is sensitive to neuroinflammatory diseases. Glia.

[21] Dollt C (2017). The shedded ectodomain of Lyve-1 expressed on M2-like tumor-associated macrophages inhibits melanoma cell proliferation. Oncotarget.

[22] Dorsam RT, Kunapuli SP (2004). Central role of the P2Y12 receptor in platelet activation. J. Clin. Investig..

[23] Vogel DY (2014). Human macrophage polarization in vitro: maturation and activation methods compared. Immunobiology.

[24] Taves MD, Gomez-Sanchez CE, Soma KK (2011). Extra-adrenal glucocorticoids and mineralocorticoids: evidence for local synthesis, regulation, and function. Am. J. Physiol. Endocrinol. Metab..

[25] Tozaki-Saitoh H (2017). P2Y12 receptors in primary microglia activate nuclear factor of activated T-cell signaling to induce C-C chemokine 3 expression. J. Neurochem..

[26] Gelosa P (2014). Microglia is a key player in the reduction of stroke damage promoted by the new antithrombotic agent ticagrelor. J. Cereb. Blood Flow. Metab..

[27] Hardy AR (2004). Reciprocal cross-talk between P2Y1 and P2Y12 receptors at the level of calcium signaling in human platelets. Blood.

[28] Ohsawa K (2007). Involvement of P2X4 and P2Y12 receptors in ATP-induced microglial chemotaxis. Glia.

[29] Suh DH (2016). P2Y12 antagonist attenuates eosinophilic inflammation and airway hyperresponsiveness in a mouse model of asthma. J. Cell. Mol. Med..

[30] Ganbaatar B (2018). Ticagrelor, a P2Y12 antagonist, attenuates vascular dysfunction and inhibits atherogenesis in apolipoprotein-E-deficient mice. Atherosclerosis.

[31] De Simone R (2010). TGF-β and LPS modulate ADP-induced migration of microglial cells through P2Y1 and P2Y12 receptor expression. J. Neurochem..

[32] Moore CS (2015). P2Y12 expression and function in alternatively activated human microglia. Neurol. Neuroimmunol. Neuroinflamm..

[33] Junger WG (2011). Immune cell regulation by autocrine purinergic signalling. Nat. Rev. Immunol..

[34] Zhang X (2018). Extracellular ADP facilitates monocyte recruitment in bacterial infection via ERK signaling. Cell. Mol. Immunol..

[35] Kawamura H, Kawamura T, Kanda Y, Kobayashi T, Abo T (2012). Extracellular ATP-stimulated macrophages produce macrophage inflammatory protein-2 which is important for neutrophil migration. Immunology.

[36] Ben Yebdri F, Kukulski F, Tremblay A, Sevigny J (2009). Concomitant activation of P2Y(2) and P2Y(6) receptors on monocytes is required for TLR1/2-induced neutrophil migration by regulating IL-8 secretion. Eur. J. Immunol..

[37] Cekic C, Linden J (2016). Purinergic regulation of the immune system. Nat. Rev. Immunol..

[38] Elliott MR (2009). Nucleotides released by apoptotic cells act as a find-me signal to promote phagocytic clearance. Nature.

[39] Cho MS (2017). Role of ADP receptors on platelets in the growth of ovarian cancer. Blood.

[40] Wang Y (2013). Platelet P2Y12 is involved in murine pulmonary metastasis. PLoS One.

[41] Gebremeskel S, LeVatte T, Liwski RS, Johnston B, Bezuhly M (2015). The reversible P2Y12 inhibitor ticagrelor inhibits metastasis and improves survival in mouse models of cancer. Int. J. Cancer.

[42] Dai M (2005). Evolving gene/transcript definitions significantly alter the interpretation of GeneChip data. Nucleic Acids Res..

[43] Subramanian A (2005). Gene set enrichment analysis: a knowledge-based approach for interpreting genome-wide expression profiles. Proc. Natl Acad. Sci. USA.

