# Supplementary material for: ADP secreted by dying melanoma cells mediates chemotaxis and chemokine secretion of macrophages via the purinergic receptor P2Y12
Source: Cell Death Dis. 2019 Oct 7;10(10):760. doi: 10.1038/s41419-019-2010-6 (PMC6779894; doi:10.1038/s41419-019-2010-6)
Supplement: Supplementary file 1 — Supplemental Data [file 41419_2019_2010_MOESM1_ESM.docx]

**Supplementary table 1. Gene expression analysis of transgenic U937 cells**. EV U937 cells (EV) and P2Y12^+^ U937 cells (P2Y12) were treated with 50 nM 2-MeSADP (ADP) for 4 h. Untreated cells were used as control (CTRL). Table shows individual intensity values of each sample (n=3).

| **Gene symbol** | **Gene name** | **EV_ctrl**  **1** | **EV_ctrl**  **2** | **EV_ctrl**  **3** | **EV_ADP**  **1** | **EV_ADP**  **2** | **EV_ADP**  **3** | **P2Y12_ctrl**  **1** | **P2Y12_ctrl**  **2** | **P2Y12_ctrl**  **3** | **P2Y12_ADP**  **1** | **P2Y12_ADP**  **2** | **P2Y12_ADP**  **3** |
| --- | --- | --- | --- | --- | --- | --- | --- | --- | --- | --- | --- | --- | --- |
| HBEGF | heparin binding EGF like growth factor | 7,3018 | 7,2998 | 7,0996 | 7,1738 | 7,1797 | 7,3457 | 7,0654 | 7,1445 | 7,0781 | 9,1719 | 9,0059 | 9,0430 |
| CXCL2 | C-X-C motif chemokine ligand 2 | 5,8330 | 6,0879 | 6,1729 | 6,0400 | 5,9316 | 6,2451 | 6,3750 | 6,1113 | 6,1172 | 7,9141 | 8,3848 | 7,6309 |
| CXCL3 | C-X-C motif chemokine ligand 3 | 4,2588 | 4,2822 | 3,8428 | 4,2139 | 3,9717 | 3,8975 | 3,5386 | 3,7393 | 3,7085 | 6,5020 | 7,1729 | 6,5313 |
| IL1B | interleukin 1 beta | 5,2139 | 5,3398 | 5,1055 | 5,4297 | 4,6924 | 5,3418 | 4,6895 | 4,6475 | 4,7432 | 9,6504 | 9,6465 | 9,6914 |
| CXCL8 | C-X-C motif chemokine ligand 8 | 6,2441 | 5,8369 | 6,2480 | 7,2637 | 6,8701 | 7,1943 | 5,6123 | 5,4854 | 5,8916 | 11,6387 | 11,6641 | 11,6016 |
| CCL3L3 | C-C motif chemokine ligand 3 like 3 | 3,8037 | 3,2969 | 4,0518 | 3,8193 | 3,5806 | 3,5303 | 3,6318 | 4,0352 | 3,6479 | 7,0088 | 6,8301 | 6,7813 |
| PPBP | pro-platelet basic protein | 2,9204 | 3,0220 | 3,1006 | 3,3677 | 3,5659 | 3,3672 | 3,0024 | 3,3022 | 3,0928 | 9,0254 | 9,0332 | 9,0586 |
| CCL20 | C-C motif chemokine ligand 20 | 4,0420 | 3,9458 | 3,9067 | 3,8306 | 4,3154 | 4,3779 | 4,0420 | 4,2031 | 3,9448 | 5,6396 | 6,6484 | 6,2090 |
| TGFB3 | transforming growth factor beta 3 | 3,8809 | 3,9839 | 4,0947 | 4,8193 | 4,8311 | 4,8818 | 3,9434 | 4,0439 | 4,1543 | 7,5498 | 7,6816 | 7,4990 |
| TNFSF8 | tumor necrosis factor superfamily member | 3,7300 | 3,7070 | 3,4395 | 4,1777 | 3,8252 | 3,9023 | 3,3608 | 3,4707 | 3,3447 | 4,9219 | 5,0977 | 5,2061 |
| TNFSF15 | tumor necrosis factor superfamily member | 3,6050 | 3,5010 | 3,4531 | 3,5068 | 3,7583 | 3,6221 | 3,3457 | 3,3423 | 3,3560 | 6,4561 | 6,5898 | 6,4639 |

**Supplementary table 2. Primer list.** Sequences of primers used for qRT-PCR.

| **Primer** | **Target sequence** |
| --- | --- |
| Hs ß-ACTIN fw | GGC ACC ACA CCT TCT ACA ATG A |
| Hs ß-ACTIN rev | TCT CCT TAA TGT CAC GCA CGA T |
| Hs CXCL2 fw | CAC AGT GTG TGG TCA ACA TTT C |
| Hs CXCL2 rev | ACA GAG GGA AAC ACT GCA TAA T |
| Hs CXCL7 fw | AAC TCC GCT GCA TGT GTA TAA |
| Hs CXCL7 rev | CCA TCC TTC AGT GTG GCT ATC |
| Hs CXCL8 fw | CCT GAT TTC TGC AGC TCT GT |
| Hs CXCL8 rev | AAA CTT CTC CAC AAC CCT CTG |
| Hs FOSL1 fw | TGA TCC ACC CAA CCC TAT CT |
| Hs FOSL1 rev | AAT GGC CTG GTC CAA TCAC |
| Hs GAPDH fw | TGC ACC ACC AAC TGC TTA GC |
| Hs GAPDH rev | GGC ATG GAC TGT GGT CAT GA |
| Hs JUN fw | CCT GAT GTA CCT GAT GCT ATG G |
| Hs JUN rev | CCT CCT GAA ACA TCG CAC TAT |
| Hs P2Y12 fw | CCA GGG TCA GAT TAC AAG AGC |
| Hs P2Y12 rev | GTT GTC GAC GGC TTG CAT TT |
